# *Lavandula angustifolia* Essential Oil as a Developmental Inhibitor of *Candida* Species and Biofilms

**DOI:** 10.3390/antibiotics15010041

**Published:** 2026-01-01

**Authors:** Vanessa Bassinello, Marcelo Fabiano Gomes Boriollo, Janaina Priscila Barbosa, Wagner Luís de Carvalho Bernardo, Mateus Cardoso Oliveira, Carlos Tadeu dos Santos Dias, Cristina Paiva de Sousa

**Affiliations:** 1 Department of Oral Diagnosis, Piracicaba Dental School, University of Campinas (FOP/UNICAMP), Limeira Avenue, 901, Piracicaba 13414-903, SP, Brazil; vanessa.bassinello@gmail.com (V.B.); janaina.priscila@hotmail.com (J.P.B.); 2 Department of Morphology and Pathology, Biotechnology Graduate Program (PPGBiotec), Center for Biological and Health Sciences (CCBS), Federal University of São Carlos (UFSCar), Washington Luís Road, Km 235, São Carlos 13565-905, SP, Brazil; wlc_bernardo@yahoo.com.br; 3 Center for Nursing and Health, State University of Southwest Bahia (UESB), José Moreira Sobrinho Avenue, Jequié 45205-490, BA, Brazil; mateus_oc1@hotmail.com; 4 Department of Exact Sciences, College of Agriculture, University of São Paulo (ESALQ/USP), 11 Pádua Dias Avenue, Piracicaba 13418-900, SP, Brazil; ctsdias@usp.br

**Keywords:** antifungal susceptibility testing, biofilms, *Candida* species, cytotoxicity, gas chromatography, germ tube, *Lavandula angustifolia* essential oil, scanning electron microscopy

## Abstract

**Background:** This research aimed to investigate the antifungal and antibiofilm action of *Lavandula angustifolia* essential oil (LaEO) against certain *Candida* species and its toxicity on human keratinocytes. **Methods:** The minimum inhibitory concentration (MIC) and sessile minimum inhibitory concentration (SMIC) of LaEO were both determined by broth microdilution assays. The influence of LaEO treatment on the ultrastructural morphology of the biofilm and germ tubes was evaluated by scanning electron microscopy (SEM) and light microscopy. In vitro cytotoxicity studies were conducted using human *HaCaT* epidermal keratinocytes. **Results:** LaEO showed fungicidal action for all *Candida* species (125–4000 μg/mL). The SMIC_>90_ (*C. albicans*) ranged between 10,000 and 20,000 μg/mL and resulted in quantitative and qualitative cellular changes. LaEO also inhibited the developmental germ tube kinetics of *C. albicans*. The 50% cytotoxic concentration (CI_50_) for *HaCaT* cells was estimated at 420 μg/mL of LaEO, resulting in a selectivity index (SI) of 0.376 to 5.753 for planktonic cells and 0.056 to 0.321 for biofilm phases. **Conclusions:** LaEO was found to have antifungal action against *Candida* species and inhibited the pathogenic morphology of *C. albicans*. Its antibiofilm effects are comparable to the antifungal agent nystatin, and it can become an important component for the development of natural products applicable to alternative and complementary medicine and dentistry.

## 1. Introduction

Some yeast species of the genus *Candida* may colonize niches in the normal microbiota of clinically healthy humans, such as the oral cavity, gastrointestinal and urinary tracts, and vaginal mucosa, without harming their hosts [1]. However, these species can become pathogenic in hosts with compromised, weakened, or violated immune systems, causing mild superficial mucosal infections to severe clinical manifestations such as oropharyngeal candidiasis, systemic infections, and candidemia [2,3,4,5,6,7]. *C. albicans* is the most medically relevant species associated with infections in humans. The pathogenicity of *Candida* species has been attributed to several virulence factors that contribute to adhesion (relative surface hydrophobicity, expression of adhesins), evasion of host defenses (high-frequency phenotypic alteration, binding of complement molecules), and the invasion and destruction of host tissues (development of germ tube and hyphae, production of secreted aspartyl protease and phospholipases). The ability to form structured biological communities within the biofilm extracellular matrix on biotic and abiotic surfaces also contributes to the antifungal treatment resistance mechanisms and host immune responses [6,8,9,10,11].

Oral candidiasis has been considered the most frequent opportunistic oral mycosis and can manifest as a variety of clinical signs and symptoms. Various local and systemic factors contribute to its development, such as the use of dental prostheses, inhaled steroid treatment, reduced salivary flow, high-carbohydrate diet, endocrine disorders (e.g., diabetes), immunosuppression (e.g., HIV-infected individuals), broad-spectrum antibiotic therapy, and nutritional deficiencies [8,12,13,14]. Long-term use of dental prostheses, whether fixed or removable, increases the risk of colonization by *Candida* species, enabling the development of prosthetic stomatitis [15,16]. Dental prostheses act as a reservoir of microorganisms and can contribute to the development of inflammation based on their construction materials and the chemical or physical methods of cleaning the prostheses [15,17,18,19].

Bacterial and fungal infections are major public health problems and are often difficult to treat due to the emergence of resistance mechanisms to the pharmaceuticals prescribed [12,13]. The main antifungal drugs used in the clinical treatment of candidiasis belong to the class of echinocandins, but azole and polyene agents can also be used with limited efficacy [20]. In addition, these drugs are not very effective against planktonic cells but even less so against the biofilms of *Candida* species, particularly *C. albicans*. Therefore, the development of new drugs for the control of these microorganisms is desperately needed [10,21]. The use of medicinal plants and herbal medicines has intensified in recent decades and continues to expand with the global demand for natural products to treat diseases [22]. Research involving essential oils as health treatments has also revealed numerous pharmacological properties and promising results for various medical therapies, including antimicrobial and anti-inflammatory actions [23].

The Lamiaceae family comprises plants rich in essential oils with natural properties valuable in natural medicine, pharmacology, biotechnology, cosmetology, and aromatherapy. About 236 genera with 6900 to 7200 species have been described in this family, the most abundant genera being *Salvia* (*n* = 900 species), followed by *Scutellaria* (*n* = 360), *Plectranthus* (*n* = 300), *Stachys* (*n* = 300), *Hyptis* (*n* = 280), *Teucrium* (*n* = 250), *Vitex* (*n* = 250), *Thymus* (*n* = 220), and *Nepeta* (*n* = 200) [24]. Currently, the genus *Lavandula* (Lamiaceae family) has 39 known species, but only *L. angustifolia* Miller, popularly known as common, true, or English lavender, has been considered as a pharmacopeia raw material [25,26,27,28,29]. Lavender (from Latin lavāre: “to wash”) is a species of indigenous aromatic medicinal plant [30] and was used by ancient societies for many purposes, from a perfume in Greece to a treatment of insanity and psychoses in Tibet. Today, it is used as an anxiolytic and sleep aid [31]. Historically, lavender has been used in traditional medicine and aromatherapy for the relief of anxiety, mental stress, insomnia, mild pain, gastrointestinal complaints, and inflammatory skin conditions, as well as for wound care and antiseptic purposes. Contemporary pharmacological studies have since associated these traditional uses with antioxidant, anti-inflammatory, sedative, spasmolytic, antimicrobial, and anticholinesterase-related effects [25,27,32,33,34]. Notably, its antimicrobial properties have only recently begun to be investigated more thoroughly, including its action against *Candida* spp. [25,32,33].

The objectives of this study were to investigate the antimicrobial action of *L. angustifolia* essential oil against *Candida* species planktonic cells, biofilms, and germ tubes, as well as its toxicity to human epidermal keratinocytes (*HaCaT* cells). Here, we report possible correlations between the biological effects of *L. angustifolia* essential oil and its phytochemical compounds.

## 2. Results

### 2.1. GC–MS of LaEO

**Figure 1:**
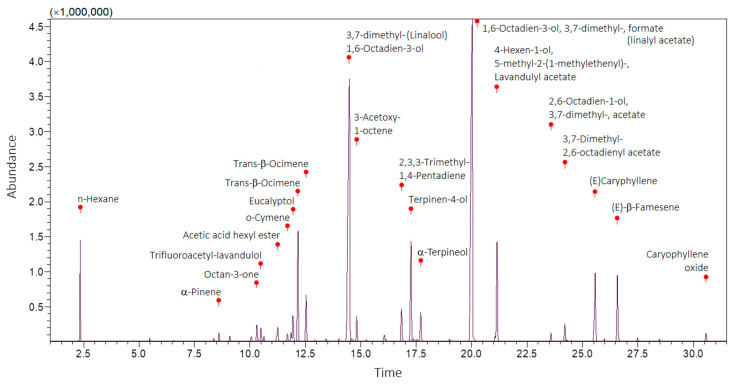
Chromatographic profiles of LaEO using gas chromatography with mass spectrometry (GC–MS), GCMS solution software version 4.20 and database (NIST 11 and FFNSC1.3). Time: *x*-axis of the gas chromatogram corresponds to retention time RT (a measure of the time taken for a solute to pass through a chromatography column) in minutes. Abundance: *y*-axis of the gas chromatogram (area of the peak) is a reflection of the amount of a specific analyte (relative distribution of compounds in the sample).

GC–MS analysis enabled the identification of 20 phytochemical constituents of LaEO under the applied analytical conditions. These correspond to the detected compounds, while additional constituents are likely present at trace levels or remain unidentified. The two main compounds were identified as 1,6-octadien-3-ol, 3,7-dimethyl- (linalool) (relative distribution: 26.99%; RT: 14.48), and 1,6-octadien-3-ol, 3,7-dimethyl-, formate (linalyl acetate) (relative distribution: 31.36%; RT: 20.02), using quality indexes > 90% (*n* = 16 compounds), >80% (*n* = 3 compounds), >70% (*n* = 1 compound), and >60% (*n* = 1 compound) (Figure 1 and Table 1).

### 2.2. Antifungal Susceptibility of LaEO

The results of antifungal susceptibility tests of LaEO revealed variable MIC values, depending on the yeast species and the method used to interpret the breakpoint (visual or colorimetric) (Table 2). The data of the two methods correlated in 5 out of 8 samples (62.5%). However, in 2 out of 8 samples (37.5%), the difference between the breakpoints was one adjacent concentration (*C. albicans* ATCC 90028 and *I. orientalis*: visual and colorimetric breakpoints of 1000 and 2000 μg/mL, respectively) while for *C. albicans* MYA-2876 the difference was two adjacent concentrations (visual and colorimetric breakpoints of 1000 and 4000 μg/mL, respectively). For the visual breakpoints, *Candida* species showed MIC values between 125 and 2000 μg/mL, where the lowest MICs were found with *M. guilliermondii* (125 μg/mL), followed by *C. albicans*, *C. glabrata*, *C. parapsilosis*, and *I. orientalis* (1000 μg/mL), and *C. lusitaniae*, and *D. rugosa* (2000 μg/mL) having the highest. Similar results were obtained with the colorimetric breakpoints, where the lowest MICs were reported for *M. guilliermondii* (125 μg/mL), followed by *C. glabrata* and *C. parapsilosis* (1000 μg/mL), *C. albicans* ATCC 90028, *C. lusitaniae*, *D. rugosa* and *I. orientalis* (2000 μg/mL), and *C. albicans* ATCC MYA-2876 (4000 μg/mL). Fungicidal activity of LaEO was observed in all *Candida* species with MFC values ranging between 500 and 4000 μg/mL (i.e., MFC ≥ MIC). The lowest MFCs of LaEO were reported for *M. guilliermondii* (500 μg/mL), followed by *C. glabrata* (1000 μg/mL), *C. albicans* ATCC 90028, *C. lusitaniae*, *C. parapsilosis*, *D. rugosa*, and *I. orientalis* (2000 μg/mL), and *C. albicans* ATCC MYA-2876 (4000 μg/mL). MIC values for FLU and AB ranged from 1 to 32 μg/mL and 0.25 to 1 μg/mL, respectively. The lowest MICs of FLU were reported for *C. albicans*, *C. lusitaniae*, *D. rugosa*, and *M. guilliermondii* (1 μg/mL), followed by *C. parapsilosis* (2 μg/mL), *C. glabrata* (8 μg/mL), and *I. orientalis* (32 μg/mL). The lowest MICs of AB were reported for *C. albicans* ATCC MYA-2876, *C. glabrata*, *C. parapsilosis*, *D. rugosa*, *I. orientalis*, and *M. guilliermondii* (0.25 μg/mL), followed by *C. lusitaniae* (0.5 μg/mL), and *C. albicans* ATCC 90028 (1 μg/mL).

### 2.3. Antibiofilm Susceptibility of LaEO

**Figure 2:**
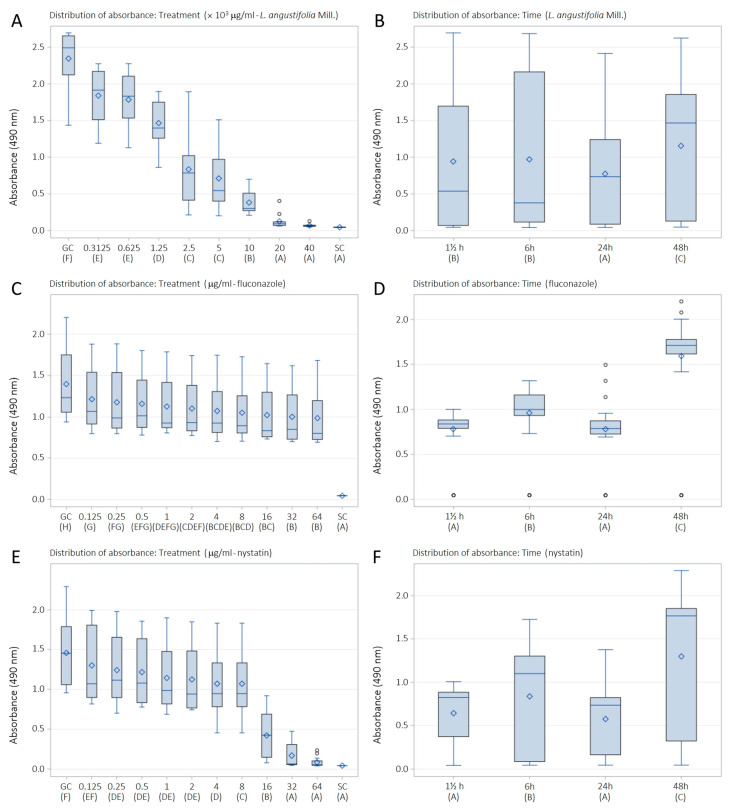
Performance of LaEO, fluconazole and nystatin on *C. albicans* biofilms developed on polystyrene and influenced by adsorbed film (BFS), using XTT reduction assays: (**A**) treatment and (**B**) exposure time for *L. angustifolia* essential oil; (**C**) treatment and (**D**) exposure time for the antifungal fluconazole; (**E**) Treatment and (**F**) exposure time for the antifungal nystatin. Concentrations of 312.5 to 40,000 μg/mL (essential oil) and 0.125 to 64 μg/mL (fluconazole and nystatin). Growth control (GC: YNB + cells). Sterility control (SC: YNB). ^A, B, C, D, E, F, G, H^—different letters indicate significantly different values (*p* < 0.05). Boxplots represent the distribution of absorbance values. Boxes indicate the interquartile range, the horizontal line represents the median, the rhombus denotes the mean value, whiskers indicate minimum and maximum values, and circles represent statistical outliers. Boxplots represent the distribution of absorbance values. Boxes indicate the interquartile range, the horizontal line represents the median, the rhombus denotes the mean value, whiskers indicate minimum and maximum values, and circles represent statistical outliers.

**Figure 3:**
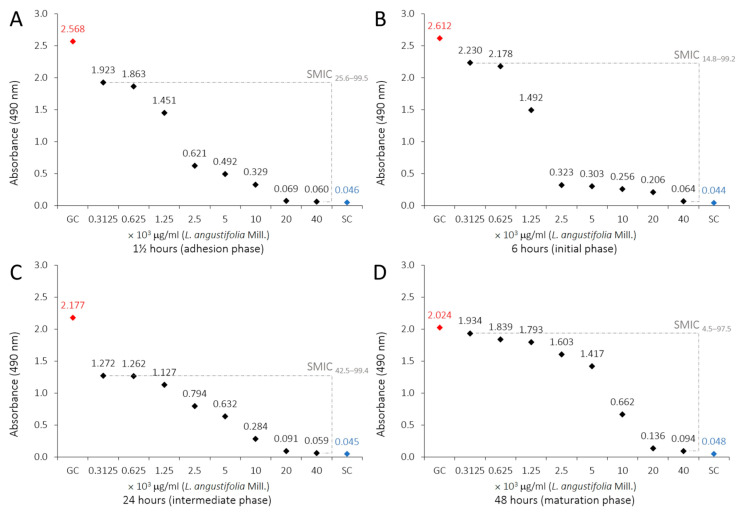
Profiles of sessile minimal inhibitory concentration (SMIC) of LaEO (312.5 to 40,000 μg/mL) on *C. albicans biofilms* (1.5, 6, 24 and 48 h) developed on polystyrene and influenced by adsorbed film (BFS): (**A**) 1.5 h (adhesion phase), (**B**) 6 h (initial phase), (**C**) 24 h (intermediate phase), and (**D**) 48 h (maturation phase). XTT reduction assays and regression analysis. Growth control (GC: YNB + cells). Sterility control (SC: YNB).

**Figure 4:**
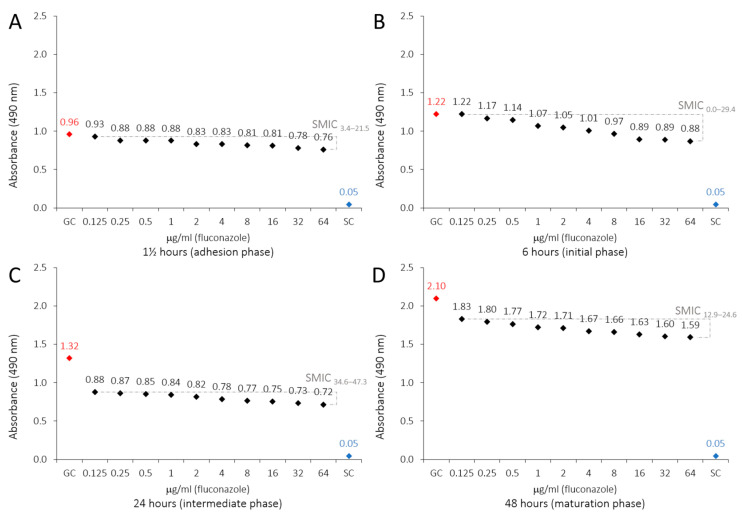
Profiles of sessile minimal inhibitory concentration (SMIC) of fluconazole (0.125 to 64 μg/mL) on *C. albicans* biofilms (1.5, 6, 24 and 48 h) developed on polystyrene and influenced by adsorbed film (BFS): (**A**) 1.5 h (adhesion phase), (**B**) 6 h (initial phase), (**C**) 24 h (intermediate phase), and (**D**) 48 h (maturation phase). XTT reduction assays and regression analysis. Growth control (GC: YNB + cells). Sterility control (SC: YNB).

**Figure 5:**
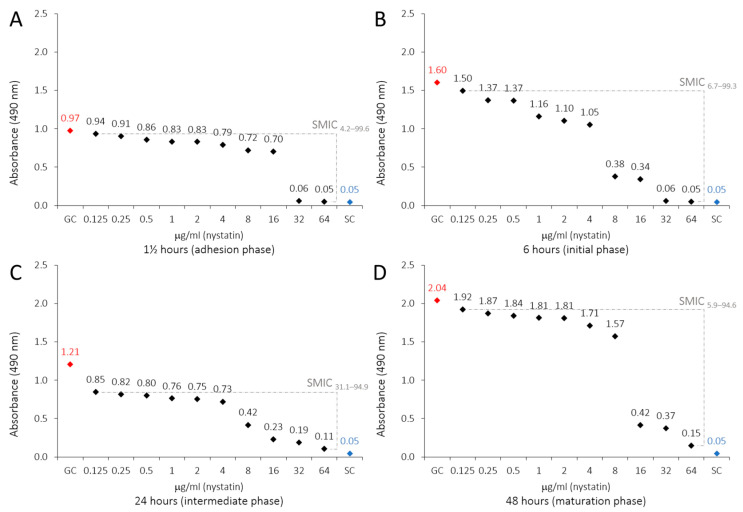
Profiles of sessile minimal inhibitory concentration (SMIC) of nystatin (0.125 to 64 μg/mL) on *C. albicans* biofilms developed on polystyrene and influenced by adsorbed film (BFS): (**A**) 1.5 h (adhesion phase), (**B**) 6 h (initial phase), (**C**) 24 h (intermediate phase), and (**D**) 48 h (maturation phase). XTT reduction assays and regression analysis. Growth control (GC: YNB + cells). Sterility control (SC: YNB).

The metabolic activity of *C. albicans* biofilms decreased with all tested LaEO concentrations, displaying that LaEO does indeed act against biofilms, with total inhibition observed at 20,000 μg/mL. FLU and NYS treatment also decreased the metabolic activity of *C. albicans* biofilms at all tested concentrations. However, FLU was not able to totally inhibit the biofilm, while NYS gave total inhibition above 32 μg/mL (Table 3, Figure 2). The biofilm sessile minimum inhibitory concentration (SMIC_90–100_) of LaEO ranged from 10,000 to 20,000 μg/mL, depending on the biofilm growth phase (Figure 3). The initial phase had the lowest SMIC, while all other phases required a higher dose. FLU did not achieve an SMIC_90–100_ at any concentration, while this was reached with NYS at 64 μg/mL (Figure 4 and Figure 5).

### 2.4. SEM of Biofilms and LaEO

**Figure 6:**
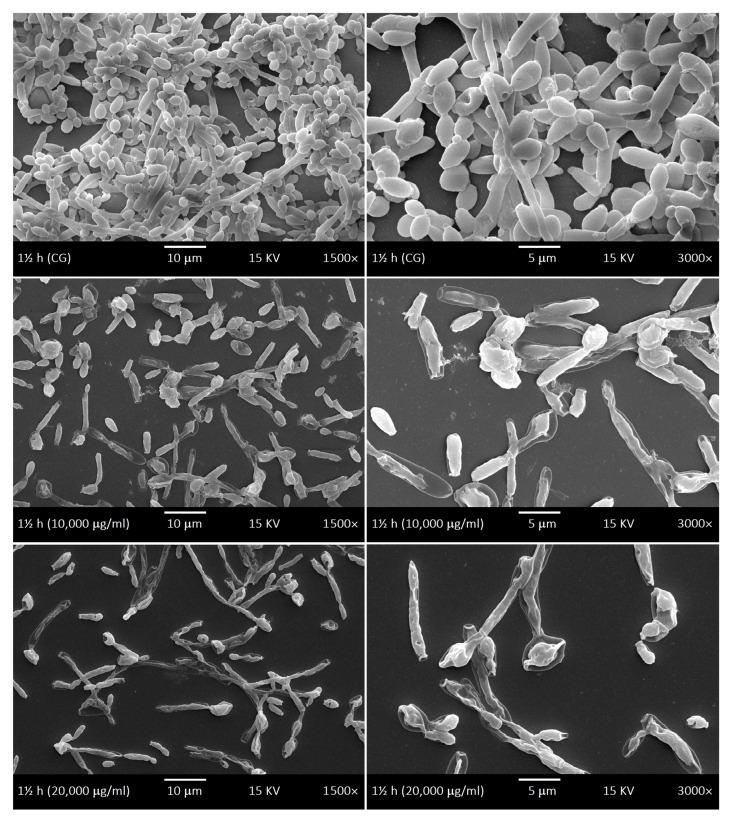
Scanning Electron Microscope (SEM) images displaying the adhesion phase (1.5 h) of the development kinetics of *C. albicans* biofilms. Control group (CG) and treatment with LaEO (10,000–20,000 μg/mL). Images generated by JEOL-JSM-5600LV (1500× and 3000× magnification, 15 kV, JEOL Ltd., Tokyo, Japan).

**Figure 7:**
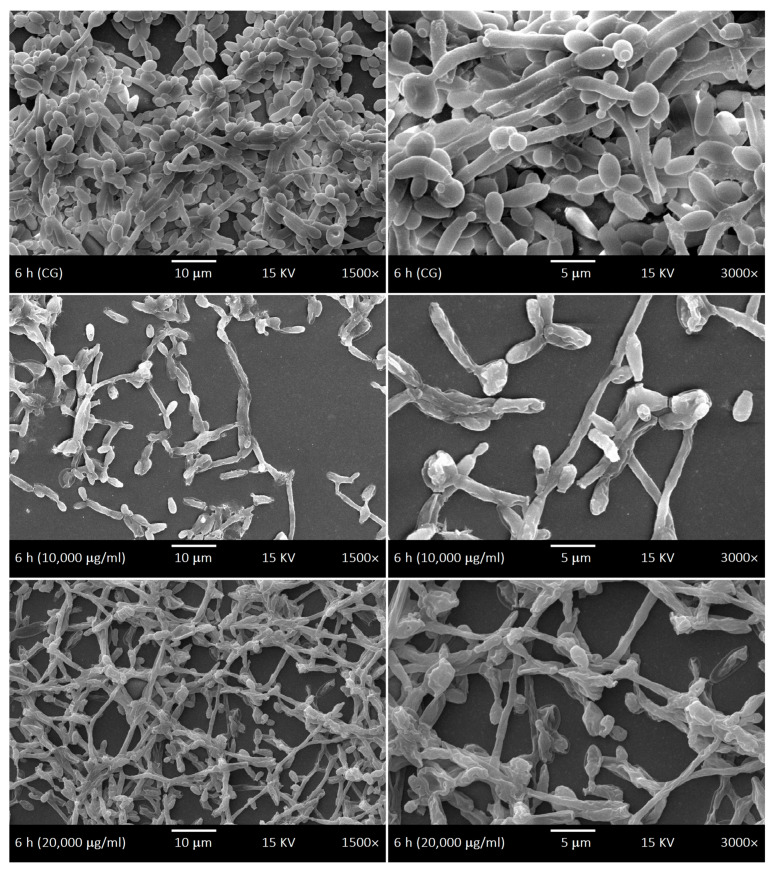
Scanning Electron Microscope (SEM) images displaying the initial phase (6 h) of the development kinetics of *C. albicans* biofilms. Control group (CG) and treatment with LaEO (10,000–20,000 μg/mL). Images generated by JEOL-JSM-5600LV (1500× and 3000× magnification, 15 kV, JEOL Ltd., Tokyo, Japan).

**Figure 8:**
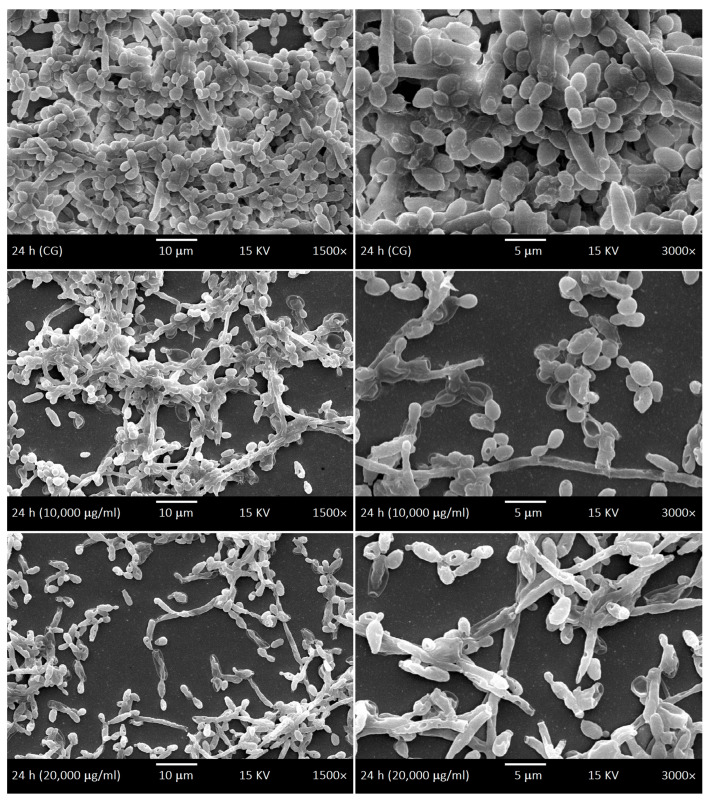
Scanning Electron Microscope (SEM) images displaying the intermediate phase (24 h) of the development kinetics of *C. albicans* biofilms. Control group (CG) and treatment with LaEO (10,000–20,000 μg/mL). Images generated by JEOL-JSM-5600LV (1500× and 3000× magnification, 15 kV, JEOL Ltd., Tokyo, Japan).

**Figure 9:**
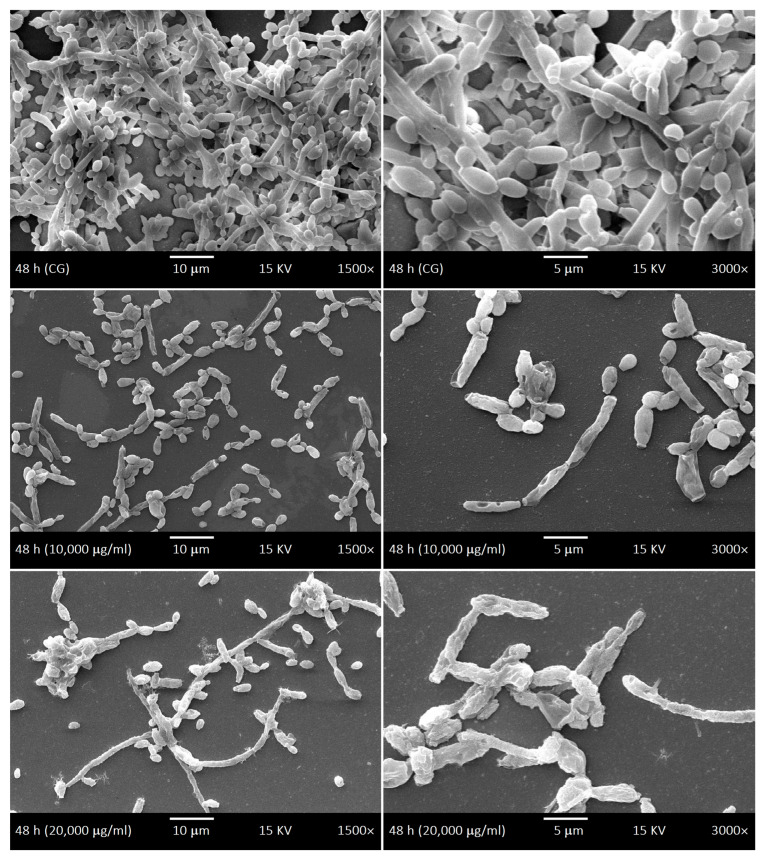
Scanning Electron Microscope (SEM) images displaying the maturation phase (48 h) of the development kinetics of *C. albicans* biofilms. Control group (CG) and treatment with LaEO (10,000–20,000 μg/mL). Images generated by JEOL-JSM-5600LV (1500× and 3000× magnification, 15 kV, JEOL Ltd., Tokyo, Japan).

SEM was used to investigate the development kinetics of *C. albicans* biofilms at different development stages with different LaEO treatments. The control biofilms of *C. albicans* showed dimorphism characteristics throughout the development phases: yeasts and hyphae, including budding and scars. The cell density gradually increased throughout the biofilm development phases (Figure 6, Figure 7, Figure 8 and Figure 9). The treatments with LaEO at concentrations of 10,000 and 20,000 μg/mL showed reduced cell adhesion (at 1.5 h of growth), reduced volume of dimorphic cell structures (yeast and hypha), plasmolysis-like morphologies, cell wall damage and consequent cytoplasmic extravasation, a significant decrease in the number of yeasts and hyphae, and a reduction and disruption of biofilm growth (6, 24, and 48 h).

### 2.5. Germ Tube Development Kinetics and LaEO

**Figure 10:**
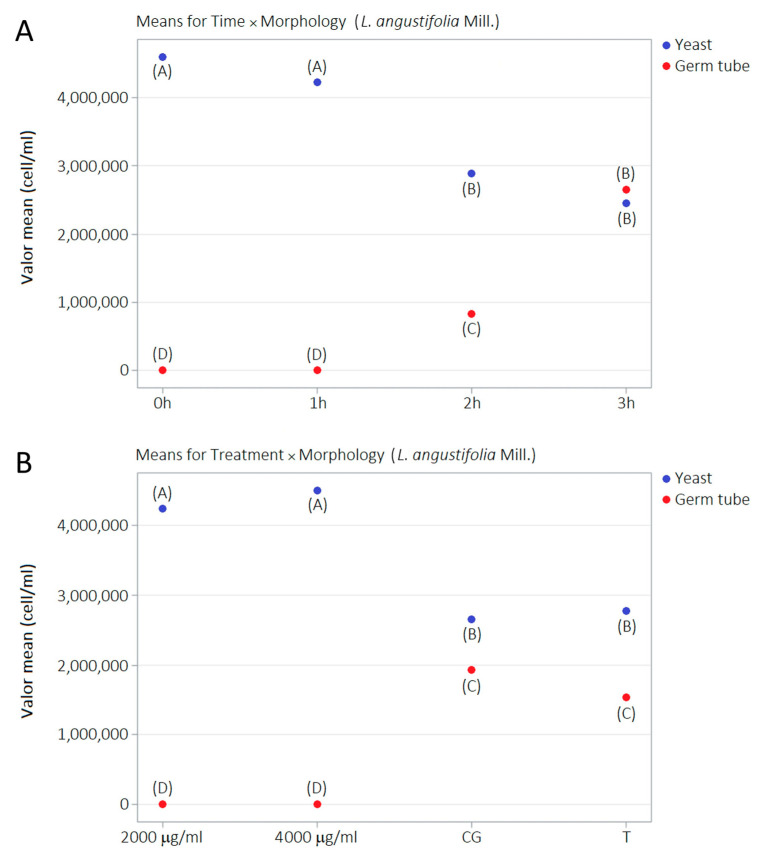
Performance of LaEO on morphological kinetics of *C. albicans* germ tubes: (**A**) means for time versus morphology and (**B**) means for treatment versus morphology. Direct counting assay using counting chambers (Petroff-Hausser Counter) and optical microscopy (magnification of 400×). Growth control (GC: YNB + BFS + cells). Tween 80^®^ (T: 0.000625%). ^A, B, C, D^—different letters indicate significantly different values (*p* < 0.05).

The number of yeast cell structures and germ tubes was statistically different (*p* < 0.05) at 0, 1, and 2 h without LaEO treatment. However, these differences among dimorphic structures were not observed at 3 h. Germ tubes emerge significantly from 2 h and increase quantitatively at 3 h and to the significant numerical detriment of yeasts from 2 h. Germ tube development was severely affected at 2000 μg/mL LaEO while it was completely abolished at 4000 μg/mL compared to the negative and Tween^®^ 80 only controls (Table 4, Figure 10).

### 2.6. Cytotoxicity of LaEO

**Figure 11:**
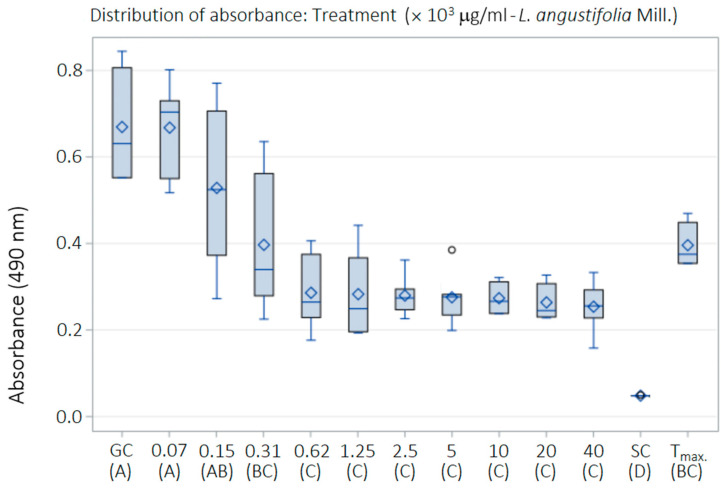
Performance of LaEO on cell cultures of normal human keratinocytes (*HaCaT*). Colorimetric assay using SRB, spectrophotometer (mean values of absorbance) and regression analysis. Concentrations of essential oil of 78.1 to 40,000 μg/mL. Growth control (GC: SRB-protein + Trizma Base). Cytotoxicity or sterility control (SC: Trizma Base). Biological effects control of Tween 80 (Tmax.: 0.05%). ^A, B, C, D^—different letters indicate significantly different values (*p* < 0.05). Boxplots represent the distribution of absorbance values. Boxes indicate the interquartile range, the horizontal line represents the median, the rhombus denotes the mean value, whiskers indicate minimum and maximum values, and circles represent statistical outliers.

**Figure 12:**
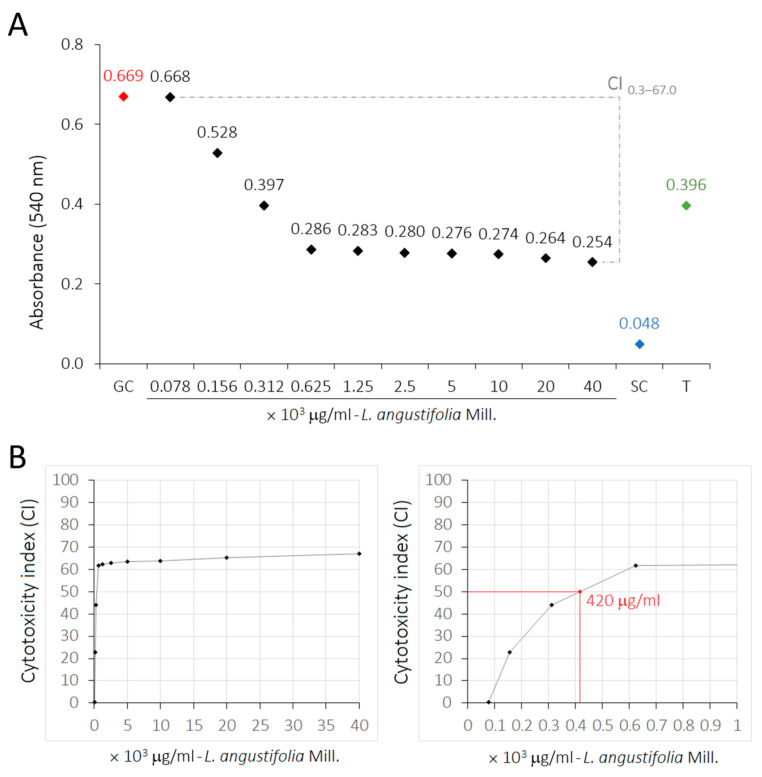
(**A**) Profiles of cytotoxicity index (CI) of LaEO on cell cultures of normal human keratinocytes (*HaCaT*). (**B**) Half-maximal inhibitory concentration (CI_50_) estimated for LaEO. Colorimetric assay using SRB, spectrophotometer (mean values of absorbance) and regression analysis (y = −0.0062x + 0.6693; R^2^ = 1). Concentrations of essential oil of 78.1 to 40,000 μg/mL. Growth control (GC: SRB-protein + Trizma Base). Cytotoxicity or sterility control (SC: Trizma Base). Biological effects control of 0.05% Tween 80 (T).

The exposure of *HaCat* cells to treatments with LaEO for 24 h resulted in a slight dose-dependent cytotoxicity (Table 5). Statistically significant differences were observed between the experimental control groups (*p* < 0.05): cell growth control (GC), cytotoxicity or sterile control (SC), and 0.05% Tween^®^ 80 (T_max_). *HaCat* cells treated with low concentrations of LaEO (78.1 and 156.2 μg/mL) exhibited results statistically equivalent to GC, indicating a lack of cytotoxicity. However, at higher concentrations of LaEO (312.5–40,000 μg/mL), some cytotoxicity was observed (Figure 11). The cytotoxicity index of 50% (CI_50_) was estimated at 420 μg/mL, with the highest dose of 40,000 μg/mL showing a CI of 67% (Figure 12).

### 2.7. Selectivity Index of LaEO

The LaEO concentrations required for 50% inhibition (half-maximal inhibitory concentration) of the planktonic cells of *Candida* species (MIC_50_), *C. albicans* biofilms (SMIC_50_), and *HaCaT* cells (CI_50_) were estimated by regression analyses based on TTZ (MIC), XTT (SMIC), and SRB (CI) assays, respectively. MIC_50_ values ranged from 73 to 1117 μg/mL depending on *Candida* species: *M. guilliermondii* (73 μg/mL), *C. glabrata* (356 μg/mL), *D. rugosa* (483 μg/mL), *C. parapsilosis* (701 μg/mL), *I. orientalis* (709 μg/mL), *C. albicans* (772 and 958 μg/mL), and *C. lusitaniae* (1117 μg/mL). However, SMIC_50_ values ranged from 1310 to 7540 μg/mL depending on the developmental stage of *C. albicans* biofilm: adhesion phase (1.5 h-SMIC_50_: 1470 μg/mL), initial phase (6 h-SMIC_50_: 1440 μg/mL), intermediate phase (24 h-SMIC_50_: 1310 μg/mL), and maturation phase (48 h-SMIC_50_: 7540 μg/mL). The CI_50_ value was estimated at 420 μg/mL. The selectivity index of LaEO ranged from 0.376 to 5.753 for the treatment of the planktonic cells of *Candida* species. A higher selectivity index of LaEO was observed in treatment against *M. guilliermondii* (SI = 5.753), followed by *C. glabrata* (SI = 1.180), *D. rugosa* (SI = 0.870), *C. parapsilosis* (SI = 0.599), *I. orientalis* (SI = 0.592), *C. albicans* (SI = 0.544 and 0.438), and *C. lusitaniae* (SI = 0.376). LaEO treatment was most selective at the intermediate phase of biofilm development (24 h-SI = 0.321), followed by the initial phase (6 h-SI = 0.292), the adhesion phase (1.5 h-SI = 0.286), and finally the maturation phase (48 h-SI = 0.056).

## 3. Discussion

The GC–MS analyses of LaEO identified two main phytochemical components: 1,6-octadien-3-ol, 3,7-dimethyl- (linalool) at 26.989%, and 1,6-octadien-3-ol, 3,7-dimethyl-, formate (linalyl acetate) at 31.363%, with many other compounds observed (see Figure 1 for details). The exact composition of the oils depends on the species and origin of the plant. Investigations have identified more than 100 molecules in lavender essential oil (*L. angustifolia*), 60 in lavender spike oil (*L. latifolia*) and more than 80 in lavandin oil (*L. intermedia*) [35,36]. The main factors that determine the phytochemical composition of essential oils and, therefore, their market value, are the type of species, variety, or hybrid; parts of the plant; cultural and climatic conditions; and the extraction method [36,37,38,39]. The most desired lavender oils come from the *L. angustifolia* species due to its high levels of linalool and linalyl acetate, while being low in camphor [40]. Terpenes and terpenoids constitute the main chemical group observed in lavender and lavandin essential oils [36].

Lavender and lavandin essential oils have traditionally been used in various manufactured products of perfumery, cosmetics, personal care (soaps, shampoos, and shower gels), home care (laundry detergent, detergents, and fabric softeners), and food flavor additives (baked goods and alcoholic and non-alcoholic beverages) [36]. Several studies have reported beneficial properties in a variety of medical and therapeutic applications, including insomnia or sleep quality improvement, alopecia, anxiety, stress, postoperative pain, and decreased excessive motor behavior [25,36,38,40,41,42,43,44,45,46,47,48,49,50,51,52,53,54,55,56]. In addition, lavender essential oils are being used in integrative medical treatments such as massage, acupuncture, and chiropractic manipulation [57].

One component of lavender essential oils, linalool, has recently been the subject of interest for its antimicrobial properties. Here, *C. albicans* strains resistant to FLU were sensitive to linalool at a dose of 256 μg/mL (the MFC). It appeared to act by affecting the cell wall and membrane integrity of the fungus by interacting with enzymes involved in their biosynthesis and maintenance [58]. Linalool is non-toxic in vitro, but it is irritating to the skin and eyes [59].

LaEO showed antifungal activity with the MIC dependent on the *Candida* species and the breakpoint interpretation method (visual or colorimetric) evaluated. Three and four breakpoints were observed in visual and colorimetric methods, respectively: 125 μg/mL (M. guilliermondii), 1000 μg/mL (*C. albicans*, *C. glabrata*, *C. parapsilosis*, and *I. orientalis*) and 2000 μg/mL (*C. lusitaniae* and *D. rugosa*) for visual breakpoints, and 125 μg/mL (*M. guilliermondii*), 1000 μg/mL (*C. glabrata* and *C. parapsilosis*), 2000 μg/mL (*C. albicans* ATCC 90028, *C. lusitaniae*, *D. rugosa*, and *I. orientalis*) and 4000 μg/mL (*C. albicans* ATCC MYA-2876) for colorimetric breakpoints. LaEO showed fungicidal action for all *Candida* species, with the exact value depending on the species and interpretation method. Our *Candida* species also showed sensitivity to antifungals FLU (1 to 32 μg/mL) and AB (0.25 to 1 μg/mL). This study showed the minimum inhibitory concentrations of the antifungals FLU and AB within the recommended limits for the quality control and reference strains [60,61,62].

This study evaluated the effects of LaEO (312.5–40,000 μg/mL) and the antifungal agents FLU and nystatin (NYS) (0.125–64 μg/mL) on cell adhesion (1.5 h) and biofilms (initial, intermediate, and maturation phases at 6, 24, and 48 h, respectively) after 24 h of exposure. LaEO showed antibiofilm activity on *C. albicans* at all concentrations tested, in a dose-dependent manner, with complete inhibition from concentrations of ≥20,000 μg/mL. It acts on each stage of biofilm development, but it significantly decreases the metabolic activity of the biofilms in the intermediate phase (24 h). The SMIC_>90_ of LaEO corresponded to 10,000 μg/mL (6 h) and 20,000 μg/mL (1.5, 24, and 48 h). As experimental controls of antifungal drugs, FLU and NYS confirmed their antibiofilm effects on *C. albicans*. FLU showed partial antibiofilm activity at all concentrations tested (except at 0.125 μg/mL at 6 h), in a dose-dependent manner. FLU was most effective at the cell adhesion (1.5 h) and intermediate phases (24 h), with SMICs between SMIC_>20_ and SMIC_<50_, depending on the stage of biofilm development. NYS revealed antibiofilm activity on *C. albicans* at all concentrations tested, in a dose-dependent manner, with complete inhibition from concentrations of ≥32 μg/mL. Again, it was effective at all stages of biofilm development but most active on cell adhesion (1.5 h) and intermediate-phase biofilms (24 h). The SMIC_>90_ of NYS corresponded to 32 μg/mL (1.5 and 6 h) or 64 μg/mL (24 and 48 h). These concentrations are higher than those for planktonic cells, which is expected as microorganisms incorporated into the biofilm show increased resistance to antibiotics and antifungals compared to their planktonic cells [63,64].

One application of this increased resistance is in dentistry with dental cavities and periodontal diseases [65]. Previous studies have suggested that components in saliva can increase *C. albicans*’ ability to develop the complex biofilms associated with prosthetic stomatitis [63]. *C. albicans* is also commonly found around dental implant sites, especially in patients with type 2 diabetes (74%) [66], and is considered the most prevalent *Candida* species in sites with peri-implant disease [67,68]. This yeast has the ability to form mature biofilms on abiotic surfaces, with implant surfaces being a suitable growth surface to produce complex and mature biofilms [69,70,71]. In addition, *C. albicans* can modulate the host’s immune response as well as interact synergistically with bacteria present at peri-implant sites, promoting increased microbial load and biofilm virulence [71].

Antifungal agents were tested to try to reduce the growth kinetics of such prosthetics. FLU and AB showed partial efficacy, depending on dosage (7.8–500 μg/mL) and timing (FLU at 24 h and AB at 1.5 h) with SMIC_50_ at 15.6 to 125 μg/mL for FLU and 7.8 μg/mL for AB [72]. However, NYS showed complete inhibition of biofilm activity by 62.5 μg/mL [11,63]. Current topical dosage guidelines recommend 20.59, 41.18 and 123.54 mg for newborns, children, and adults, respectively, with a treatment period of one to four weeks [73].

Similarly, LaEO in this study showed inhibition of biofilm activity at all developmental stages but at higher concentrations (SMIC_90_ of 10,000–20,000 μg/mL), suggesting its potential use in pharmaceutical and medical products as well as more everyday products such as mouthwashes and toothpastes. Under SEM, quantitative and qualitative cellular changes were observed, such as reduced cell volume, cell wall damage leading to cytoplasm leakage, and decreases in the number of yeast cells and hyphae. Linalool and linalyl acetate, the main components of LaEO, have previously been shown to disrupt cell membranes, which would partially explain our observations [74]. Therefore, LaEO has the potential to be a natural active agent in products aimed at controlling oral *Candida* infections in patients with dental implants.

The antifungal activity of LaEO observed in this study, with MIC values ranging from 125 to 4000 µg/mL against *Candida* species, is consistent with the strain-dependent efficacy widely reported for lavender-derived essential oils. Such variability reflects both differences in fungal susceptibility and the inherent compositional complexity of essential oils. As reported in the literature, lavender oils (*L. × intermedia* ‘Budrovka’ and *L. angustifolia*) enriched in oxygenated monoterpenes, particularly linalool (53.97–57.10%) and linalyl acetate (9.83–11.56%), exhibited pronounced inhibitory and fungicidal activity against *Candida* spp., including *C. albicans*, *C. glabrata*, *C. kefyr*, *C. krusei* and *C. tropicalis* (MIC values ranging from 0.10 to 0.75 mg/mL and MFC values between 0.2 and 1.0 mg/mL) [25]. In agreement with these findings, comparable antifungal activity against *C. albicans* has been described for lavender oils containing balanced proportions of linalyl acetate (36.7%), linalool (31.4%) and terpinen-4-ol (14.9%), with reported MIC values around 3 mg/mL [33]. Further supporting this observation, lavender oil rich in linalool (32.75%) and linalyl acetate (43.13%) inhibited the growth of *C. albicans* at concentrations between 0.125 and 2% and exhibited fungicidal activity at concentrations ranging from 0.5 to 4%. In this context, *C. albicans* ATCC 3153 showed MIC and MFC values of 0.5% and 1%, respectively, while clinical isolates from vaginal infections (*n* = 22) displayed MIC and MFC values of 0.69 ± 0.31% and 1.1 ± 0.45%, and isolates from oropharyngeal infections (*n* = 28) showed MIC and MFC values of 1.04 ± 0.35% and 1.8 ± 0.9%, respectively [32]. The fungicidal and fungistatic properties of the oil depended on the exposure time and its concentration, where 2% essential oil eliminated 99% and 100% of cells in 5 and 15 min, respectively, but required 30 min at 0.5% [32]. In addition, similar concentrations were found to be effective against *C. albicans* strains found in clinical sputum samples, even in strains resistant to FLU [75].

LaEO has been shown to increase the effectiveness of clotrimazole on vaginal *C. albicans* infections [76] along with ciprofloxacin against *S. aureus* and chloramphenicol against *P. aeruginosa* [33]. These studies further strengthen the potential clinical use of LaEO in a variety of infections and conditions as long as the oil contains high levels of linalool and linalyl acetate (above 29.93% and 27.55%, respectively [77]. Liposomes have also been suggested to increase treatment efficacy by protecting the compounds against oxidation and increasing their solubility. Protecting the oil components from oxidation is particularly important, as their method of action includes the production of reactive oxygen species as well as influencing biofilm gene regulation [78]. LaEO is also likely to inhibit infection spread by inhibiting germ tube formation at 4 mg/mL, as germ tubes are a critical factor in the progression and spread of the fungus [32]. Therefore, by controlling the formation of germ tubes, infections can be more easily controlled, resulting in less damage to structures [79,80,81,82].

LaEO was shown here to show in vitro cytotoxic effects in normal human keratinocytes (human *HaCaT* epidermal keratinocytes) at levels above 625 μg/mL, but dosages below 312.5 μg/mL were considered statistically non-toxic. These values were influenced by the levels of linalool and linalyl acetate, and therefore, the therapeutic use of LaEO needs to account for varying chemical compositions due to geographical origin, forms of cultivation, and oil extraction processes. The cytotoxic effects contributed to a less-than-ideal SI as, while shown to be comparable to NYS treatment, its SI in biofilms ranged from 0.056 to 0.321. Therefore, future LaEO treatments may display a certain degree of cytotoxicity to mammalian cells as well as to the *Candida* species being treated. Pioneering investigations have systematically assessed the cytotoxic effects of lavender essential oil (0.016–2% *v*/*v*) and its principal constituents, linalyl acetate (0.008–1.020% *v*/*v*) and linalool (0.005–0.700% *v*/*v*), using (i) human fibroblasts (153BR), (ii) human normal dermal fibroblasts (HNDF), and (iii) simian virus 40–transformed human dermal microvascular endothelial cells (HMEC-1) [83]. For lavender oil, mean cell viabilities ranging from 80 to 100% were observed across all three cell lines at concentrations ≤0.125% (*v*/*v*), whereas a 50% growth inhibition was estimated between 0.169 and 0.195% (*v*/*v*). A pronounced decline in cell viability occurred at concentrations ≥0.250% (*v*/*v*). Linalyl acetate exhibited markedly higher cytotoxicity toward HNDF and 153BR fibroblasts, with 50% growth inhibition estimated at 0.028% (*v*/*v*) and 0.031% (*v*/*v*), respectively. In HMEC-1 endothelial cells, a comparable inhibitory effect was observed only at the highest tested concentration, approximately 0.360% (*v*/*v*). In contrast, linalool demonstrated a statistically analogous response profile (supported by a confirmed linear relationship) to that of lavender oil across all cell lines. This correspondence is consistent with its relative abundance in the oil, as gas chromatography analyses identified linalool and linalyl acetate as comprising approximately 35% and 51% of the oil, respectively. Notably, cell viability declined significantly at linalool concentrations ≥0.088% (*v*/*v*), corresponding by analogy to ≥0.250% (*v*/*v*) lavender oil. Collectively, these findings indicate that linalyl acetate exhibits greater cytotoxic potential than the whole essential oil, suggesting that the acetate functional group confers higher toxicity relative to the alcohol moiety of linalool, the primary bioactive component of lavender oil. Membrane disruption was proposed as a plausible underlying mechanism of action. Taken together, these observations underscore the necessity for cautious use of lavender oil and its constituents, particularly in topical applications, where highly diluted formulations are strongly advised [83]. Taken together, these findings highlight the need for systematic cytotoxic and toxicological investigations, including dose–response, exposure time, and formulation-dependent effects, to better define the therapeutic window and translational potential of LaEO and its major constituents.

## 4. Materials and Methods

### 4.1. Essential Oil Source

The *L. angustifolia* essential oil (LaEO) was commercially obtained (Terra-Flor Indústria e Comércio de Aromaterápicos Ltd., Alto Paraíso de Goiás, GO, Brazil; color: pale yellow to amber yellow; density: 0.883 g/cm^3^; refractive index: 1.461; optical rotation: −7.3; lot no. 21184; manufactured on 2 August 2021) and stored according to the manufacturer’s recommendations (at 4 °C). The phytochemical screening was carried out at the Biochemistry and Instrumental Analysis Multiuser Center (CBAI), Department of Agroindustry, Food and Nutrition, College of Agriculture, University of São Paulo (ESALQ/USP).

### 4.2. GC–MS Analysis

LaEO was analyzed by gas chromatography coupled to mass spectrometry (GC–MS) and a mass spectral database (GCMS-QP 2010 Plus, Shimadzu Corporation, Tokyo, Japan). Here, 100 nL was injected into a cross-bond dimethyl polysiloxane low-polarity phase column at 50 °C. The temperature was then increased to 200 °C at a rate of 4 °C/min before increasing the temperature further to 240 °C at 10 °C/min and remaining there for seven minutes. Readings were taken at 230 °C at 70 eV and 40–500 m/z. The data obtained (retention time and area at GC–MS total ion chromatogram—TIC) were processed using the GCMS solution software version 4.20 (Shimadzu Corp., Tokyo, Japan). The identities of the compounds were obtained with their similarity to library data (NIST 11 and FFNSC1.3) and LRI calculation (Linear Retention Index) from the injection series of C7–C30 alkanes.

### 4.3. Yeasts and Growth Conditions

This study used yeast strains belonging to the Biobank of the Dental School of Piracicaba, University of Campinas (FOP/UNICAMP, Piracicaba, SP, Brazil), kept in cryogenic systems. The yeast species used in the essential oil susceptibility assays (planktonic cells) were as follows: *C. albicans* (ATCC 90028; Isolation source: blood; CLSI: Reference strain), *C. albicans* (ATCC MYA-2876; Isolation source: clinical specimen), *C. glabrata* (IZ07; Zymotechnic Institute, College of Agriculture, University of São Paulo—ESALQ/USP, Piracicaba, SP, Brazil), *C. lusitaniae* (ATCC 42720; Isolation source: blood of patient with myelogenous leukemia), *C. parapsilosis* (ATCC 22019; Type strain; Isolation source: case of sprue; CLSI: Quality Control strain), *D. rugosa* (ATCC 10571; Type strain, deposited as *Candida rugosa* (Anderson) Diddens et Lodder; Isolation source: feces), *I. orientalis* (ATCC 6258; Type strain of *Candida krusei*, deposited as *Candida krusei* (Castellani) Berkhout; Isolation source: sputum of patient with bronchomycosis; CLSI: Quality Control strain), and *M. guilliermondii* (ATCC 6260; Type strain of *Candida guilliermondii*, deposited as *Monilia guilliermondii* (Castellani) Castellani et Chalmers; Isolation source: sputum from patient with bronchomycosis).

In addition, *C*. *albicans* (ATCC MYA-2876) was used to assess the potential effect of the LaEO on biofilm and germ tube development kinetics and the ultrastructural morphology of biofilms. The yeasts were cultured in SDA agar at 35 °C for 24–48 h under aerobic conditions before the tests [11,63,72,84,85].

### 4.4. Antifungal Susceptibility Tests

The MIC of LaEO was determined by broth microdilution, using CLSI guidelines [60,61,62], with some adaptations. The tests were performed in triplicate systems using 96-well microplates containing RPMI 1640 medium buffered with MOPS (Sigma-Aldrich, Merck KGaA, Darmstadt, Germany) and the microbial inoculum supplemented with LaEO (final volume of 100 μL/well). The concentrations of LaEO used in the test ranged from 15.6 μg/mL to 32,000 μg/mL (Tween^®^ 80 from 0.00002% to 0.05%) of RPMI 1640 culture medium. Yeast cultures were adjusted to a transmittance of 79.5–83.2% at a wavelength of 530 nm using a spectrophotometer (Genesys 10 UV-Visible Spectrophotometer, Thermo Electron Corporation, Vantaa, Finland) in sterile saline solution. The yeast suspension was vortexed for 15 s and diluted at a 1:50 ratio in RPMI 1640 medium, followed by a new 1:20 dilution to give a final content of 1–5 × 10^3^ cfu/mL. During the assays, 50 μL aliquots of each working inoculum (50% of well volume) were placed in the microdilution wells, containing 50 μL/well of RPMI 1640 culture medium and the differing concentrations of the essential oil to be tested. Afterward, the microdilution plates were incubated at 37 °C for 24 h under aerobic conditions. The antifungals amphotericin B (AB) (Sigma-Aldrich, Merck KGaA, Darmstadt, Germany) and fluconazole (FLU) (Sigma-Aldrich, Merck KGaA, Darmstadt, Germany) were also used as experimental effective antifungal controls and quality controls (QC). Standard solutions of these antifungals were prepared, sterilized by filtration, and stored at –70 °C. The concentrations of AB and FLU used in the antimicrobial tests ranged from 0.0313 to 16 μg/mL and from 0.125 to 64 μg/mL of RPMI 1640 medium, respectively. The interpretation of breakpoints was performed visually according to the CLSI guidelines. In addition, colorimetric assays were performed in order to determine the MIC of LaEO, based on TTZ reduction by mitochondrial enzymes (Sigma-Aldrich, Merck KGaA, Darmstadt, Germany) [11,63,72,84,85].

### 4.5. Minimum Fungicidal Concentration

The minimum fungicidal concentration (MFC) of LaEO was determined as described previously [72,84,85,86,87,88]. For each yeast strain, 100 μL aliquots of the total well volume corresponding to the ≥ MIC value were homogenized with a pipette, spread on Petri dishes containing SDA media, and incubated at 35 °C for 24–48 h under aerobic conditions. MFC was the lowest concentration of essential oil able to eliminate ≥99.9% of the final inoculum.

### 4.6. Antibiofilm Tests

The effects of LaEO on the development kinetics of *C. albicans* biofilms, specifically their metabolic bioactivities, were determined as previously described [63,89,90,91,92,93,94].

#### 4.6.1. Culture of Planktonic Yeasts

Yeast species of *C. albicans*, freshly developed on SDA medium, were cultivated in 50 mL Falcon tubes containing 10 mL of YNB broth (Invitrogen, Thermo Fisher Scientific, São Paulo, SP, Brazil) under aerobic conditions at 37 °C for 24 h at 100 rpm (Orbital Shaking Mini Incubator, Nova Técnica Ltd.a., Piracicaba, SP, Brazil). Afterward, these cultures were centrifuged at 9200× *g* (Eppendorf do Brasil Ltd.a., São Paulo, SP, Brazil) for 5 min at 4 °C and washed in 20 mL sterile PBS buffer solution, repeated three times. The yeast cells were then adjusted to a transmittance of 10% using a spectrophotometer at a wavelength of 600 nm before the biofilm assays.

#### 4.6.2. Adsorb Films

FBS (fetal bovine serum) was used as a preconditioning system capable of generating adsorb films on the developmental surfaces of *C. albicans* biofilms. FBS (Gibco, Thermo Fisher Scientific, São Paulo, SP, Brazil) was inactivated at 56 °C for 30 min and stored at −20 °C until further use. Prior to the assays, 100 μL aliquots of FBS were aseptically dispensed into sterile wells (96-well microplates, flat-bottom, Corning, Corning, NY, USA) and incubated overnight at 37 °C under slight agitation (Incubator TE-4200, Tecnal Equipamentos Ltd., Piracicaba, SP, Brazil). Then, the FBS was removed, and each well of the microplates was carefully washed once with sterile PBS buffer solution.

#### 4.6.3. Biofilm Control

For the development of *C. albicans* biofilms (growth controls), 100 μL aliquots of cell cultures (1 × 10^7^ cfu/mL) were transferred to microplates containing adsorb films of FBS and statically incubated at 37 °C for 1.5 h (adhesion phase). Soon after, non-adherent yeast cells (planktonic) were carefully removed by washing with PBS solution once. Next, 100 μL of YNB broth supplemented with glucose (100 mM) was added to each microplate well before being incubated at 37 °C, shaken at 75 rpm (Thermo Shaker Dry Block, Nova Instruments, São Paulo, SP, Brazil), for either 6 h (initial phase), 24 h (intermediate phase), or 48 h (maturation phase). Afterward, non-adherent yeast cells (planktonic) were carefully removed by washing with PBS solution once. For each condition, a negative control of the adsorbed films and YNB without yeast cells was used. All assays were performed in triplicate.

#### 4.6.4. Treatment Groups of Biofilms

The effect of LaEO on *C. albicans* biofilms (SMIC) was assessed by the broth microdilution method, partially based on the CLSI guidelines [60,61,62,63]. Prior to the tests, the standard solutions (2×) of the essential oil (90.840 mL of YNB broth supplemented with glucose + 100 μL of Tween^®^ 80 + 9.060 mL of LaEO) were serially diluted in YNB broth supplemented with glucose in order to produce the different concentrations to be tested. All the developmental stages of the biofilms (1.5, 6, 24, and 48 h), including sterility controls, were treated with LaEO solutions (100 μL aliquots) at concentrations ranging from 312.5 μg/mL to 40,000 μg/mL (Tween^®^ 80 ranged from 0.0004% to 0.05%) for 24 h at 37 °C, agitated at 75 rpm. All the concentrations tested were above the MIC for planktonic cells. FLU and nystatin (NYS) (Sigma-Aldrich, Merck KGaA, Darmstadt, Germany) were also used as experimental antifungal controls [63]. The concentrations of these antifungals used in the *C. albicans* antibiofilm assays ranged from 0.125 μg/mL to 64 μg/mL (in YNB broth supplemented with glucose).

#### 4.6.5. Bioactivity Analysis

The metabolic activities of the *C. albicans* biofilms (treatment and control groups) were monitored by the semiquantitative XTT reduction assay. Prior to the assay, freshly prepared menadione solution (10 mM crystalline menadione solution in acetone) was added to XTT-saturated solutions (500 μg/mL in PBS buffer solution) (Merck KGaA, Darmstadt, Germany) to achieve a concentration of 1 μM menadione. Then, 100 μL of this solution was added to previously cultured biofilms as described above. After incubation for 3 h at 37 °C, 80 μL aliquots of each sample were transferred to sterile microplates, and their formazan products were quantified by spectrophotometry (490 nm).

### 4.7. Ultrastructural Morphology of Biofilms

The effects of LaEO on the ultrastructural morphologies of *C. albicans* biofilms were observed by using SEM technologies as previously reported [72,85], with some adaptations. The culture of planktonic yeasts, preconditioning system, and development of control biofilms were performed as described above, using glass slides (Ø 15 mm, Bioland Scientific, LLC., Paramount, CA, USA) and 24-well cell culture plates (Corning^®^ Costar^®^, Merck KGaA, Darmstadt, Germany). Biofilms at each development stage were exposed to LaEO (300 μL) for 24 h at 37 °C, shaken at 75 rpm. The concentrations tested ranged from 10,000 to 20,000 μg/mL (Tween^®^ 80 ranged from 0.0125% to 0.025%), corresponding to SMIC_90_ > SMIC*_n_* ≥ SMIC_80_ and SMIC_100_ ≥ SMIC*_n_* ≥ SMIC_90_ values, respectively, for the majority of biofilm development times, and the lowest concentration statistically compatible with sterility control and the first concentration statistically different from the sterile control, respectively. The SMIC_≤100_ of the LaEO, FLU, and NYS against *C. albicans* biofilms was calculated by regression analysis, using the absorbance mean values. After the treatment period, the slides were carefully washed with PBS solution twice and fully dried at room temperature. Afterward, these glass slides were fixed using a 2% glutaraldehyde solution in PBS for 30 min, dehydrated by washing in 50%, 70%, 90%, and absolute ethanol for 10 min twice each, and dried at room temperature. Finally, the slides were metallized with gold at 20 Ω for 120 s (Desk V—Standard, Delton Vacuum, LLC, Moorestown, NJ, USA, JOEL United States, Inc.) and analyzed using SEM (JSM-5600 LV JEOL, Tokyo, Japan) at 15 KV and 1500× and 3000× magnification.

### 4.8. Germ Tube Induction Assays

The effects of LaEO on the development kinetics of germ tubes were determined according to previously reported methodologies [95,96,97], with some adaptations [63], using *C. albicans* and inactivated FBS.

#### 4.8.1. Yeast Culture

Yeasts were cultivated in 50 mL of YEPD medium (yeast extract 1% *w*/*v*, peptone 2% *w*/*v*, and D-glucose 2% *w*/*v*) at 37 °C for 18 h, shaken at 150 rpm. The cells were then centrifuged at 3000× *g* for 5 min and washed twice in sterile PBS solution. These cells were finally resuspended in 5 mL of PBS solution and adjusted to a McFarland standard no. 1.0 (2–10 × 10^6^ cfu/mL) or corresponding to a transmittance of 10% using a spectrophotometer at wavelengths of 530 nm.

#### 4.8.2. Treatment Throughout Germ Tube Development Kinetics

The yeast cells suspended in PBS solution were vortexed for 15 s, diluted 1:10 (2–10 × 10^5^ cfu/mL) in FBS (with or without LaEO), and incubated at 37 °C for up to 3 h. The treatments used LaEO at concentrations of 0, 2000, and 4000 μg/mL (with Tween^®^ 80 at 0.000625, 0.0003125, and 0.000625%, respectively). An additional negative control without LaEO and Tween^®^ 80 was also used. The concentrations of LaEO tested represented the ≤MIC values (planktonic cells), depending on the interpretation method of breakpoints (visually or using the colorimetric assays). Yeast cells and germ tubes were evaluated and quantified by direct counting using light microscopy at 400× magnification (Carl Zeiss Axiostar Plus Microscópio Binocular, Carl Zeiss AG, Oberkochen, Germany) and with the aid of a hemocytometer at 4 different times (0, 1, 2, and 3 h).

### 4.9. In Vitro Cytotoxicity Tests

#### 4.9.1. Cell Culture

The human *HaCaT* epidermal keratinocyte cell line [98], belonging to the Biobank of the School of Dentistry of Piracicaba, State University of Campinas (FOP/UNICAMP), was used in the cytotoxicity study of LaEO, as previously reported [72,99,100,101,102,103]. The keratinocytes were cultured in culture flasks (TPP Techno Plastic Products AG, Trasadingen, Switzerland) containing 5 mL of RPMI 1640 medium (SIGMA, Merck KGaA, Darmstadt, Germany), supplemented with 10% inactivated FBS (SIGMA, Merck KGaA, Darmstadt, Germany), 0.1% 2-mercaptoethanol (SIGMA, Merck KGaA, Darmstadt, Germany), 1% antibiotic (Penicillin-Streptomycin, SIGMA, Merck KGaA, Darmstadt, Germany), and 1% amino acid solution (SIGMA, Merck KGaA, Darmstadt, Germany). Initially, the cells were monitored under standard culture conditions at 37 °C with the media being renewed every two days in an atmosphere containing 5% CO_2_ (Incubator, SANYO Electric Co., Ltd., Osaka, Japan), until they reached a confluent growth above 90%. Then, the cells were treated with 5 mL of trypsin-containing solution (Gibco™, Thermo Fisher Scientific, Waltham, MA, USA) for 5 min before transferring these treated cells to Falcon tubes containing 5 mL of RPMI 1640 medium, and centrifuged at 3000 rpm (Eppendorf, Hamburg, Germany) for 5 min at 20 °C. The cell pellets were resuspended in 10 mL of a solution of 1:5 RPMI 1640 to culture medium (Petroff-Hausser Counting Chamber, Horsham, PA, USA), and adjusted to a concentration of 5 × 10^4^ cells/mL.

For the assays, 100 μL aliquots of the cell suspension were distributed into 96-well cell culture microplates (NEST Biotechnology Co., Ltd., Wuxi, China). After seeding, cells were incubated for 24 h at 37 °C in a 5% CO_2_ atmosphere to promote cell adhesion and homeostasis. Afterward, the culture media were carefully aspirated and replaced with 100 μL of culture medium supplemented with varying concentrations of LaEO (from 40,000 μg/mL to 78.1 μg/mL). Keratinocytes were exposed to LaEO for 24 h at 37 °C and in a 5% CO_2_ atmosphere. Sterile culture medium was used as a negative and of total cell death. The keratinocytes were also exposed to a solution of Tween^®^ 80 (Sigma-Aldrich, Merck KGaA, Darmstadt, Germany), previously diluted in culture medium at a final concentration of 0.05% (i.e., its maximum concentration used in the dilution of the LaEO), to evaluate its potential biological effect. Keratinocytes not exposed to LaEO were used as a cell growth control. Each series consisted of 6 replicates corresponding to the different growth conditions described above.

#### 4.9.2. Sulforhodamine B Assay

Cell viability [99] was assessed by sulforhodamine B (SRB) assays [101]. After 24 h incubation, the post-culture media were carefully removed, and the cells were washed with PBS solution. To fix the cells, 50 μL aliquots of 10% trichloroacetic acid were added to each cell well before being incubated for 1 h at 4 °C. After fixation, the cells were washed five times with distilled water and dried at room temperature overnight [101]. Subsequently, 50 μL aliquots of a freshly prepared 0.04% SRB solution (Aldrich, Merck KGaA, Darmstadt, Germany) with 1% acetic acid were added to each well and the plates were kept in the dark for 1 h at 4 °C. Excess dye was then removed from each well by washing them four times with a 1% acetic acid solution and dried for 40 min at room temperature. Cell-associated SRB was solubilized (150 μL/well) in a 10 mM Trizma Base solution, pH 10.5 (Sigma, Merck KGaA, Darmstadt, Germany) for 30 min, shaken at 200 rpm before being measured at 540 nm (Genesys 10 UV-Visible Spectrophotometer, Thermo Electron Corporation, Vantaa, Finland).

### 4.10. Selectivity Index

The SI of LaEO was calculated with the following formulas: SI = CI_≤50_ ÷ MIC_≤50_ and SI = CI_≤50_ ÷ SMIC_≤50_ viability [104,105,106]. The SI > 2 and SI < 2 values correspond to the presence and absence of compound selectivity, respectively. Therefore, the higher the *HaCat*: *Candida* spp. ratio (CI_≤50_:MIC_≤50_ or CI_≤50_:SMIC_≤50_), the higher the selectivity of LaEO and, consequently, the lower the cytotoxicity on mammalian cells under the conditions of the in vitro assay [104,105,106,107].

## 5. Conclusions

In conclusion, this study characterized the main phytochemical compounds in LaEO and demonstrated its anti-*Candida* spp. action by inhibiting biofilm metabolism and germ tube formation. However, we also found some potential cytotoxic effects of this essential oil on mammalian cells. This inhibitory action was shown for a number of species (*C. albicans*, *C. glabrata*, *C. lusitaniae*, *C. parapsilosis*, *D. rugosa*, *I. orientalis*, and *M. guilliermondii*) with the exact MIC and MFC values being species-dependent. These MIC values were approximately 5 to 20 times lower compared to the SMIC values gained for the biofilms, with LaEO most efficacious against the intermediate biofilm phase. The effectiveness of LaEO treatment was comparable to NYS treatment but at much higher active component concentrations (around 625 times higher). In addition, LaEO treatment affected the ultrastructural morphological characteristics of *C. albicans* biofilms and planktonic cells (i.e., in both dimorphic shapes: yeasts and hyphae), including reduced microbial population density, decreased cell volume, plasmolysis-like mechanisms, and cell wall damage followed by cytoplasmic extravasation. LaEO was also found to inhibit germ tube formation, thereby reducing the virulence and spread of *C. albicans*. However, LaEO was suggested to be toxic to mammalian cells at the levels required to inhibit *C. albicans*, especially when cells are contained in biofilms. Thus, this essential oil can become an important target for natural product development applicable to alternative and complementary medicine and dentistry if its limitations are observed.

## Figures and Tables

**Table 1:** Phytochemical composition of *L. angustifolia* essential oil (LaEO) identified by GC–MS analysis.

No.	Phytochemical Compound	RT	LRI	Score	Peak Area	Peak Area	Putative Identification
		(Min)	(Exp.)	(%)	(Counts)	(%)	(Library)
1	*n*-Hexane	2.34	–	93	2,468,433	3.24	NIST 11
2	α-Pinene	8.60	816	95	242,734	0.32	FFNSC 1.3
3	Octan-3-one	10.31	846	96	590,837	0.77	FFNSC 1.3
4	Trifluoroacetyl-lavandulol	10.49	849	67	540,636	0.71	NIST 11
5	Acetic acid hexyl ester	11.25	862	97	626,875	0.82	FFNSC 1.3 + NIST 11
6	*o*-Cymene	11.69	870	95	252,386	0.33	FFNSC 1.3 + NIST 11
7	Eucalyptol	11.94	874	96	1,075,399	1.41	FFNSC 1.3 + NIST 11
8	Trans-β-Ocimene	12.16	878	95	4,682,461	6.14	FFNSC 1.3 + NIST 11
9	Trans-β-Ocimene	12.53	885	89	1,884,034	2.47	NIST 11
10	1,6-Octadien-3-ol, 3,7-dimethyl-(Linalool)	14.48	921	97	20,589,825	26.99	FFNSC 1.3 + NIST 11
11	3-Acetoxy-1-octene	14.81	928	96	818,961	1.07	FFNSC 1.3 + NIST 11
12	2,3,3-Trimethyl-1,4-Pentadiene	16.83	968	74	1,350,399	1.77	NIST 11
13	Terpinen-4-ol	17.26	977	93	4,912,866	6.44	FFNSC 1.3 + NIST 11
14	α-Terpineol	17.71	985	89	766,231	1.00	FFNSC 1.3 + NIST 11
15	1,6-Octadien-3-ol, 3,7-dimethyl-, formate (linalyl acetate)	20.02	1036	98	23,927,019	31.36	FFNSC 1.3 + NIST 11
16	4-Hexen-1-ol, 5-methyl-2-(1-methylethenyl)-, Lavandulyl acetate	21.14	1061	98	4,372,447	5.73	FFNSC 1.3 + NIST 11
17	2,6-Octadien-1-ol, 3,7-dimethyl-, acetate, (Z)-	23.58	1119	93	279,904	0.37	FFNSC 1.3 + NIST 11
18	3,7-Dimethyl-2,6-octadienyl acetate	24.20	1135	94	717,837	0.94	FFNSC 1.3
19	(E)-Caryphyllene	25.57	1169	98	3,032,598	3.98	FFNSC 1.3
20	(E)-β-Famesene	26.57	1195	84	2,791,995	3.66	NIST 11
21	Caryophyllene oxide	30.56	1306	92	366,199	0.48	FFNSC 1.3

Putative identification of all compounds was achieved by comparison of electron impact (EI, 70 eV) mass spectra with FFNSC v1.3 and NIST 11 libraries, combined with calculation of linear retention indices (LRI). RT, retention time (min); LRI (exp.), linear retention index experimentally calculated relative to a homologous series of *n*-alkanes (C7–C30) on a low-polarity capillary column. (–), LRI could not be reliably calculated due to elution outside the alkane calibration range. Similarity score (%) refers to the degree of match between experimental and reference mass spectra. Peak area (%) represents the relative abundance of each compound, calculated from the normalized total ion chromatogram (TIC) peak areas. Compounds presenting lower similarity scores (<80%) were assigned lower confidence.

**Table 2:** Numerical and correlation profiles of the antimicrobial susceptibility tests of LaEO and selectivity index involving *Candida* species.

*Candida* Species	*L. angustifolia* Essential Oil (μg/mL)					SA (μg/mL)	
	Visual		TTZ Assays		SI	FLU	AB
	MIC	MFC	MIC_100_	MIC_50_		MIC	MIC
*C. albicans* (ATCC 90028)	1000	2000	2000	958	0.438	1	1
*C. albicans* (ATCC MYA-2876)	1000	4000	4000	772	0.544	1	0.25
*C. glabrata* (IZ07)	1000	1000	1000	356	1.180	8	0.25
*C. lusitaniae* (ATCC 42720)	2000	2000	2000	1117	0.376	1	0.5
*C. parapsilosis* (ATCC 22019)	1000	2000	1000	701	0.599	2	0.25
*D. rugosa* (ATCC 10571)	2000	2000	2000	483	0.870	1	0.25
*I. orientalis* (ATCC 6258)	1000	2000	2000	709	0.592	32	0.25
*M. guilliermondii* (ATCC 6260)	125	500	125	73	5.753	1	0.25

SA, standard antifungal; FLU, fluconazole; AB, amphotericin B; MIC, minimal inhibitory concentration; MFC, minimal fungicidal concentration; MIC_50_, LaEO concentration required for 50% inhibition of *Candida* species planktonic cells (MIC_50_ values were estimated by dose–response regression analysis of absorbance data derived from the broth microdilution assay, rather than being directly observed); SI, selectivity index, calculated as the ratio between CI_50_ and MIC_50_ values; CI_50_, cytotoxicity index corresponding to the half-maximal inhibitory concentration on *HaCat* cells (estimated at 420 μg/mL).

**Table 3:** XTT reduction assay measurements of LaEO, fluconazole and nystatin activity on *C. albicans* biofilms formed on polystyrene and conditioned by BFS.

Treatment	1½ h		6 h		24 h		48 h	
	Mean ± SD	SMIC	Mean ± SD	SMIC	Mean ± SD	SMIC	Mean ± SD	SMIC
*L. angustifolia* Mill. essential oil (μg/mL)								
GC ^F^	2.568 ± 0.219	0.0	2.612 ± 0.064	0.0	2.177 ± 0.271	0.0	2.024 ± 0.594	0.0
312.5 ^E^	1.923 ± 0.230	25.6	2.230 ± 0.045	14.8	1.272 ± 0.072	42.5	1.934 ± 0.059	4.5
625 ^E^	1.863 ± 0.239	28.0	2.178 ± 0.092	16.9	1.262 ± 0.152	43.0	1.839 ± 0.015	9.3
1250 ^D^	1.451 ± 0.230	44.3	1.492 ± 0.305	43.6	1.127 ± 0.230	49.3	1.793 ± 0.128	11.7
2500 ^C^	0.621 ± 0.185	77.3	0.323 ± 0.096	89.1	0.794 ± 0.022	64.9	1.603 ± 0.338	21.3
5000 ^C^	0.492 ± 0.083	82.4	0.303 ± 0.101	89.8	0.632 ± 0.091	72.5	1.417 ± 0.150	30.7
10,000 ^B^	0.329 ± 0.063	88.8	0.256 ± 0.041	91.7	0.284 ± 0.046	88.9	0.662 ± 0.039	68.8
20,000 ^A^	0.069 ± 0.001	99.1	0.206 ± 0.172	93.6	0.091 ± 0.031	97.9	0.136 ± 0.079	95.3
40,000 ^A^	0.060 ± 0.011	99.5	0.064 ± 0.004	99.2	0.059 ± 0.003	99.4	0.094 ± 0.030	97.5
SC ^A^	0.046 ± 0.002	100	0.044 ± 0.004	100	0.045 ± 0.001	100	0.048 ± 0.001	100
RA (SMIC)	y = −0.0252x + 2.5677		y = −0.0257x + 2.6119		y = −0.0213x + 2.1769		y = −0.0198x + 2.0241	
Fluconazole (μg/mL)								
GC ^H^	0.958 ± 0.019	0.0	1.222 ± 0.022	0.0	1.318 ± 0.178	0.0	2.095 ± 0.099	0.0
0.125 ^G^	0.927 ± 0.082	3.4	1.221 ± 0.079	0.0	0.878 ± 0.075	34.6	1.830 ± 0.043	12.9
0.25 ^FG^	0.877 ± 0.046	8.9	1.172 ± 0.151	4.2	0.865 ± 0.083	35.7	1.795 ± 0.076	14.6
0.5 ^EFG^	0.876 ± 0.004	9.0	1.144 ± 0.033	6.6	0.852 ± 0.071	36.7	1.766 ± 0.039	16.1
1 ^DEFG^	0.876 ± 0.015	9.0	1.067 ± 0.123	13.1	0.842 ± 0.051	37.5	1.721 ± 0.075	18.2
2 ^CDEF^	0.834 ± 0.039	13.6	1.046 ± 0.046	14.9	0.817 ± 0.039	39.4	1.711 ± 0.026	18.7
4 ^BCDE^	0.832 ± 0.038	13.8	1.007 ± 0.046	18.2	0.785 ± 0.098	42.0	1.668 ± 0.094	20.8
8 ^BCD^	0.814 ± 0.021	15.7	0.967 ± 0.034	21.6	0.765 ± 0.081	43.5	1.657 ± 0.115	21.4
16 ^BC^	0.811 ± 0.058	16.1	0.893 ± 0.139	27.9	0.754 ± 0.012	44.4	1.631 ± 0.015	22.7
32 ^B^	0.779 ± 0.101	19.6	0.889 ± 0.073	28.2	0.735 ± 0.041	45.9	1.604 ± 0.012	24.0
64 ^B^	0.761 ± 0.055	21.5	0.875 ± 0.095	29.4	0.717 ± 0.022	47.3	1.590 ± 0.149	24.6
SC ^A^	0.046 ± 0.000	100	0.045 ± 0.000	100	0.045 ± 0.000	100	0.045 ± 0.000	100
RA (SMIC)	y = −0.0091x + 0.9575		y = −0.0118x + 1.2218		y = −0.0127x + 1.3178		y = −0.0205x + 2.0951	
Nystatin (μg/mL)								
GC ^F^	0.974 ± 0.027	0.0	1.601 ± 0.073	0.0	1.207 ± 0.145	0.0	2.041 ± 0.217	0.0
0.125 ^EF^	0.935 ± 0.030	4.2	1.495 ± 0.286	6.7	0.847 ± 0.038	31.1	1.923 ± 0.060	5.9
0.25 ^DE^	0.905 ± 0.016	7.5	1.372 ± 0.129	14.7	0.818 ± 0.126	33.6	1.869 ± 0.098	8.6
0.5 ^DE^	0.860 ± 0.018	12.3	1.366 ± 0.082	15.0	0.799 ± 0.025	35.1	1.838 ± 0.017	10.1
1 ^DE^	0.832 ± 0.016	15.3	1.163 ± 0.040	28.1	0.762 ± 0.066	38.3	1.811 ± 0.077	11.5
2 ^DE^	0.829 ± 0.054	15.6	1.103 ± 0.112	31.9	0.753 ± 0.010	39.2	1.810 ± 0.065	11.6
4 ^D^	0.791 ± 0.088	19.8	1.054 ± 0.136	35.1	0.726 ± 0.271	41.5	1.711 ± 0.166	16.5
8 ^C^	0.722 ± 0.040	27.2	0.380 ± 0.240	78.3	0.422 ± 0.388	67.7	1.572 ± 0.091	23.4
16 ^B^	0.702 ± 0.023	29.3	0.341 ± 0.164	80.7	0.228 ± 0.168	84.4	0.416 ± 0.444	81.3
32 ^A^	0.062 ± 0.004	98.1	0.058 ± 0.011	98.9	0.188 ± 0.144	87.8	0.373 ± 0.102	83.4
64 ^A^	0.048 ± 0.005	99.6	0.051 ± 0.004	99.3	0.106 ± 0.078	94.9	0.149 ± 0.083	94.6
SC ^A^	0.046 ± 0.000	100	0.045 ± 0.000	100	0.045 ± 0.000	100	0.046 ± 0.000	100
RA (SMIC)	y = −0.0093x + 0.9744		y = −0.0156x + 1.6006		y = −0.0116x + 1.2071		y = −0.02x + 2.0408	

BFS, bovine fetal serum; SD, standard deviation; SMIC, sessile minimal inhibitory concentration (SMIC); RA, regression analysis (R^2^ = 1). Concentrations of 3.125 to 40,000 μg/mL (essential oil) and 0.125 to 64 μg/mL (fluconazole and nystatin). Growth control (GC: YNB + cells). Sterility control (SC: YNB). Regression analysis (RA). ^A, B, C, D, E, F, G, H^ Significantly different (*p* < 0.05).

**Table 4:** Incidence of yeasts and germ tubes (cells/mL) of *C. albicans* exposed to LaEO treatment for 0–3 h.

Morphology	Treatment	Time			
		0 h ^A^	1 h ^A^	2 h ^B^	3 h ^B^
Yeast	CG ^B^	4.73 ± 0.231	4.40 ± 0.477	1.22 ± 0.603	0.27 ± 0.252
	2000 μg/mL ^A^	4.43 ± 0.451	4.28 ± 0.493	3.97 ± 0.161	4.27 ± 0.161
	4000 μg/mL ^A^	4.27 ± 0.284	4.67 ± 0.225	4.63 ± 0.306	4.47 ± 0.237
	Tween 80 ^B^	4.97 ± 0.189	3.58 ± 0.404	1.75 ± 0.458	0.80 ± 0.346
Morphology	Treatment	Time			
		0 h ^D^	1 h ^D^	2 h ^C^	3 h ^B^
Germ tube	CG ^C^	0.0 ± 0.0	0.0 ± 0.0	1.67 ± 0.605	6.10 ± 2.311
	2000 μg/mL ^D^	0.0 ± 0.0	0.0 ± 0.0	0.0 ± 0.0	0.017 ± 0.029
	4000 μg/mL ^D^	0.0 ± 0.0	0.0 ± 0.0	0.0 ± 0.0	0.0 ± 0.0
	Tween^®^ 80 ^C^	0.0 ± 0.0	0.0 ± 0.0	1.67 ± 0.333	4.48 ± 0.506

Values are expressed as mean ± standard deviation (*n* × 10^6^). CG (control group), Tween^®^ 80 (0.000625%) and SD (standard deviation). Means with the same letter (A, B, C or D) are not significantly different (*p* < 0.05).

**Table 5:** Performance of LaEO on cell cultures of normal human keratinocytes (*HaCaT*). Colorimetric assay using SRB, spectrophotometer (mean values of absorbance) and regression analysis.

Treatment—*L. angustifolia* Essential Oil	Mean ± SD	CI
GC ^A^	0.669 ± 0.127	0.0
78.1 μg/mL (0.000097% Tween 80) ^A^	0.668 ± 0.110	0.3
156.2 μg/mL (0.00019% Tween 80) ^AB^	0.528 ± 0.190	22.7
312.5 μg/mL (0.00039% Tween 80) ^BC^	0.397 ± 0.165	44.0
625 μg/mL (0.00078% Tween 80) ^C^	0.286 ± 0.090	61.8
1250 μg/mL (0.00156% Tween 80) ^C^	0.283 ± 0.103	62.3
2500 μg/mL (0.00312% Tween 80) ^C^	0.280 ± 0.048	62.9
5000 μg/mL (0.00625% Tween 80) ^C^	0.276 ± 0.063	63.5
10,000 μg/mL (0.125% Tween 80) ^C^	0.274 ± 0.039	63.8
20,000 μg/mL (0.025% Tween 80) ^C^	0.264 ± 0.042	65.4
40,000 μg/mL (0.05% Tween 80) ^C^	0.254 ± 0.059	67.0
SC ^D^	0.048 ± 0.001	100.2
T_max_ ^BC^	0.396 ± 0.052	44.1

SRB, Sulforhodamine B; SD, standard deviation; CI, cytotoxicity index (regression analysis: y = −0.0062x + 0.6693; R^2^ = 1). Concentrations of essential oil of 78.1 to 40,000 μg/mL. Growth control (GC: SRB-protein + Trizma Base). Sterility control (SC: Trizma Base). T_max_ (0.05% Tween 80). ^A, B, C, D^ Significantly different (*p* < 0.05).

## Data Availability

Dataset available on request from the authors.

## Abbreviations

The following abbreviations are used in this manuscript:

AB	Amphotericin B
ANOVA	Analysis of Variance
ATCC	American Type Culture Collection
CFU	Colony-Forming Units
CI_50_	Cytotoxicity Index 50
FBS	Fetal Bovine Serum
FLU	Fluconazole
GC–MS	Gas Chromatography–Mass Spectrometry
*HaCaT*	Human Adult Low Calcium High Temperature Keratinocytes
LaEO	*Lavandula angustifolia* Essential Oil
LRI	Linear Retention Index
MFC	Minimum Fungicidal Concentration
MIC	Minimum Inhibitory Concentration
MIC_50_	Inhibitory Concentration for 50% Reduction
MOPS	3-(N-Morpholino)propanesulfonic Acid
NYS	Nystatin
PBS	Phosphate-Buffered Saline
QC	Quality Control
RPMI	Roswell Park Memorial Institute Medium
SDA	Sabouraud Dextrose Agar
SEM	Scanning Electron Microscopy
SI	Selectivity Index
SMIC	Sessile Minimum Inhibitory Concentration
SMIC_50_	Sessile Inhibitory Concentration for 50% Reduction
SRB	Sulforhodamine B
TIC	Total Ion Chromatogram
TTZ	Triphenyltetrazolium Chloride
UV	Ultraviolet
XTT	2,3-Bis(2-methoxy-4-nitro-5-sulphophenyl)-5-[(phenylamino)carbonyl]-2H-tetrazolium Hydroxide
YEPD	Yeast Extract Peptone Dextrose
YNB	Yeast Nitrogen Base
